# Sex-Specific Embryonic Gene Expression in Species with Newly Evolved Sex Chromosomes

**DOI:** 10.1371/journal.pgen.1004159

**Published:** 2014-02-13

**Authors:** Susan E. Lott, Jacqueline E. Villalta, Qi Zhou, Doris Bachtrog, Michael B. Eisen

**Affiliations:** 1 Department of Molecular and Cell Biology, University of California, Berkeley, Berkeley, California, United States of America; 2 Howard Hughes Medical Institute, University of California, Berkeley, Berkeley, California, United States of America; 3 Department of Integrative Biology, University of California, Berkeley, Berkeley, California, United States of America; Fred Hutchinson Cancer Research Center, United States of America

## Abstract

Sex chromosome dosage differences between females and males are a significant form of natural genetic variation in many species. Like many species with chromosomal sex determination, Drosophila females have two X chromosomes, while males have one X and one Y. Fusions of sex chromosomes with autosomes have occurred along the lineage leading to *D. pseudoobscura* and *D. miranda*. The resulting neo-sex chromosomes are gradually evolving the properties of sex chromosomes, and neo-X chromosomes are becoming targets for the molecular mechanisms that compensate for differences in X chromosome dose between sexes. We have previously shown that *D. melanogaster* possess at least two dosage compensation mechanisms: the well- characterized MSL-mediated dosage compensation active in most somatic tissues, and another system active during early embryogenesis prior to the onset of MSL-mediated dosage compensation. To better understand the developmental constraints on sex chromosome gene expression and evolution, we sequenced mRNA from individual male and female embryos of *D. pseudoobscura* and *D. miranda*, from ∼0.5 to 8 hours of development. Autosomal expression levels are highly conserved between these species. But, unlike *D. melanogaster*, we observe a general lack of dosage compensation in *D. pseudoobscura* and *D. miranda* prior to the onset of MSL-mediated dosage compensation. Thus, either there has been a lineage-specific gain or loss in early dosage compensation mechanism(s) or increasing X chromosome dose may strain dosage compensation systems and make them less effective. The extent of female bias on the X chromosomes decreases through developmental time with the establishment of MSL-mediated dosage compensation, but may do so more slowly in *D. miranda* than *D. pseudoobscura*. These results also prompt a number of questions about whether species with more sex-linked genes have more sex-specific phenotypes, and how much transcript level variance is tolerable during critical stages of development.

## Author Summary

Many animals have sex-specific combinations of chromosomes. In humans, for example, females have two X chromosomes while males have one X and one Y. In most species with XX:XY systems, the Y chromosome is degenerate and gene-poor while the X encodes a large number of functional genes. A variety of systems have evolved to ensure that males with one X chromosome and females with two X chromosomes have the same gene expression level for X-linked genes. The vinegar fly *D. melanogaster* has at least two dosage compensation systems: one that acts early in development, and another active in later stages. In this paper, we determine expression levels for thousands of genes in male and female embryos at different developmental stages in two species, *D. pseudoobscura* and *D. miranda*, that have unusually large fractions of their genomes in X or X-like chromosomes. We show that dosage compensation is established slowly during embryogenesis, and that in these species, dosage compensation appears to be absent in early development. This may be due to a lineage-specific loss or gain of compensation mechanism, or possibly because the machinery of dosage compensation cannot effectively handle the increased demand in these species.

## Introduction

Differing dosage of sex chromosomes is one of the most significant forms of natural genetic variation that animals with genetic sex determination face. In *Drosophila*, like humans, females have two X chromosomes, while males have one X and one Y chromosome. Thus, roughly half of the population (males) is hemizygous for the entire X chromosome.

Throughout evolutionary history, many different mechanisms have evolved to compensate transcription for this difference in sex chromosome dosage [1],[2]. Eutherian mammals transcriptionally inactivate one of the two X chromosomes in females, the nematode *Caenorhabditis elegans* downregulates gene expression from both X chromosomes in XX hermaphrodites, while *Drosophila* upregulate expression from the single X chromosome in males [3]–[5]. Thus, uncompensated sex chromosomal dosage is clearly a problem for many lineages with XY sex chromosomes, solvable in many different ways. Note that ZW systems, where females are the heterogametic sex, often lack chromosome-wide dosage compensation, though gene-specific compensation mechanisms have evolved in these systems [6],[7].

The canonical *Drosophila* dosage compensation mechanism involves the formation of the MSL protein complex (or dosage compensation complex; DCC), which consists of at least 5 proteins: male specific lethal- 1, 2, and 3 (MSL-1, MSL-2, MSL-3), males absent on the first (MOF), and maleless (MLE), and two non-coding RNAs *RNA on the X 1* and *2* (*rox-1*, *rox-2*). MOF is a histone acetylase that catalyzes the acetylation of lysine 16 of histone H4 (H4K16ac) across the X chromosome in males. The DCC binds the X chromosome at particular sites (known as high affinity or chromatin entry sites), and is thought to spread to adjacent actively transcribed genes. Through this process, the chromatin landscape of the X chromosome is altered, to produce a twofold upregulation of transcription of the single male X (see [8]–[10] for review).

The establishment of MSL-mediated dosage compensation occurs after the onset of zygotic transcription. A handful of zygotic genes are transcribed prior to and during stage 4 (mitotic cycles 10–13, [11],[12]), but widespread zygotic activation of transcription occurs during mitotic cycle 14 (stage 5), at the time when the blastoderm is undergoing cellularization. The male-specific component of the DCC, *msl-2*, is not observed until late in stage 5 [13]–[15], and the earliest observation of the H4K16ac established by the DCC is at stage 9 [13],[14], leaving a gap of several hours of development between the onset of zygotic transcription and the establishment of MSL-mediated dosage compensation.

As there are many important processes occurring during this period of development (such as mitotic cycling, segmentation along the anterior-posterior axis, the formation of germ layers along the dorsal-ventral axis, cellularization of the blastoderm, and gastrulation) but no canonical dosage compensation mechanism, we set out in a previous study to characterize gene expression in female and male *Drosophila melanogaster* embryos during this period of development [15]. In order to do so, we developed techniques to sequence mRNA from single embryos, allowing for precise staging and acquisition of sex-specific data. In that study, focusing on stages 3–5 (spanning the onset of zygotic transcription), we found that about half of zygotically expressed genes on the X chromosome had roughly equal transcript levels between female and male embryos. Thus, there was some form of incomplete early zygotic dosage compensation, by unknown mechanism(s), before the onset of MSL-mediated dosage compensation, consistent with earlier genetic studies on a single gene active during this period [16],[17].

This left many questions unanswered, including several evolutionary ones: Does early zygotic dosage compensation vary across *Drosophila* species? If so, how did early zygotic dosage compensation evolve? Is it dependent on gene content or age of the X chromosome? And, additionally, since our previous study in *D. melanogaster* ended at the end of stage 5, could we, by extending the timecourse, observe the onset of MSL-mediated dosage compensation? What would that look like? And how would it compare to early zygotic dosage compensation?

The complex evolutionary history of sex chromosomes in *Drosophila* provides a basis for addressing many of these questions. Fusions of ancestral sex chromosomes to autosomes have happened numerous times in the evolutionary history of *Drosophila*, leading to the creation of new (neo) sex chromosomes [18]. As *Drosophila* males lack meiotic recombination, male-specific neo-sex chromosomes (neo-Ys) are completely sheltered from recombination and degenerate over time due to the decreased efficacy of natural selection on a non-recombining chromosome [19]. As genes on the neo-Y degenerate, their homologous copies on the neo-X become hemizygous in males and potential targets for dosage compensation.

**Figure 1:**
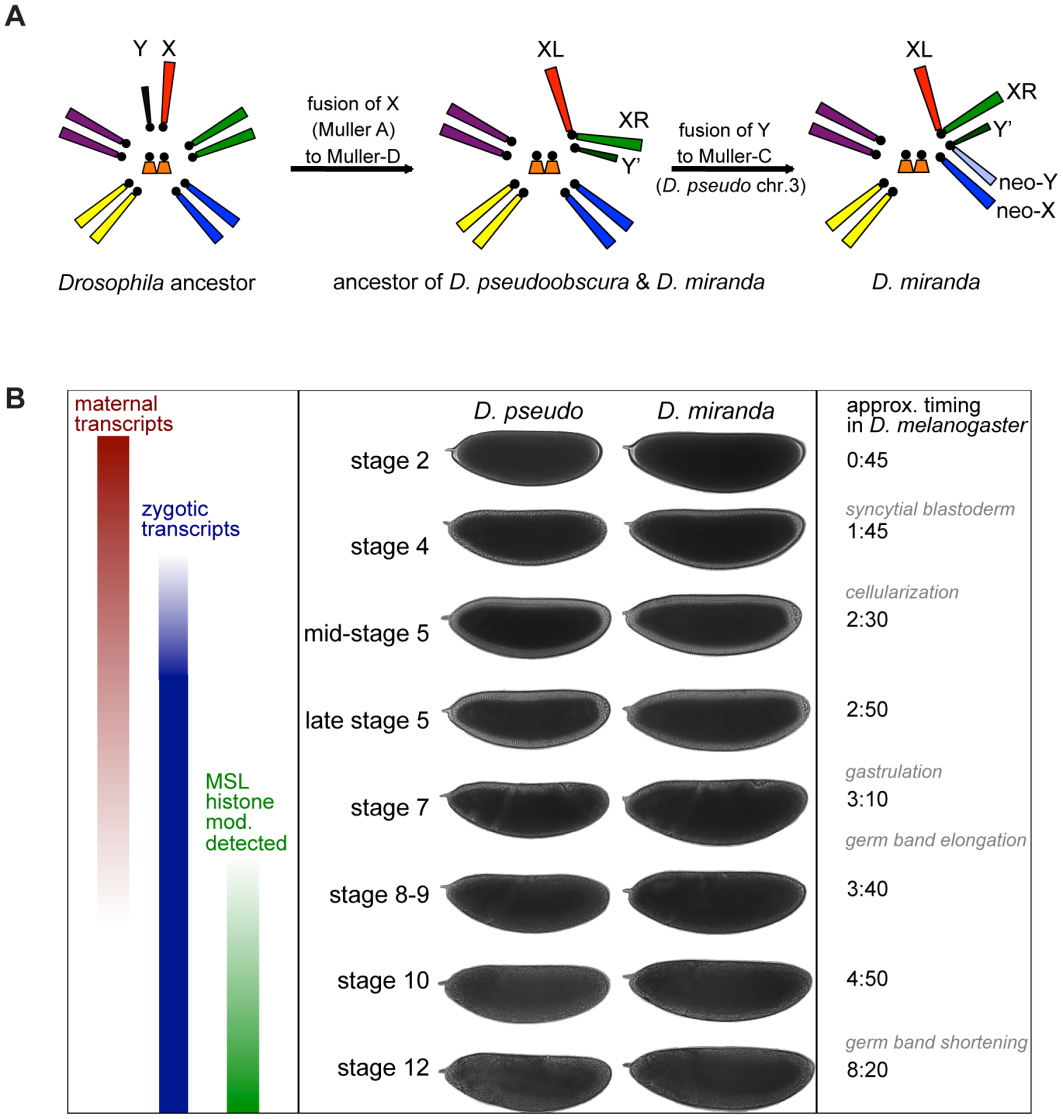
Karyotype and timecourse. A) Karyotype of a *Drosophila* ancestor, *D. pseudoobscura* and *D. miranda* ancestor (and current karyotype of *D. pseudoobscura*), and *D. miranda*. B) Important transcriptional events in the early embryo, examples of embryo staging for the two species, based on morphology, and timing of these events in *D. melanogaster* at 25°C. MSL histone modification (mod.) refers to observation of the histone modification H4K16ac, mediated by the MSL complex, by antibody staining in *D. melanogaster* [13],[14].

Here we focus on two closely related species, *Drosophila pseudoobscura* and *Drosophila miranda*, which carry neo-sex chromosomes of different evolutionary ages (Figure 1A). In the lineage leading to both *D. pseudoobscura* and *D. miranda*, the ancestral X chromosome (XL in these species) fused with an autosome (Muller element D, or 3L in *D. melanogaster*), to create chromosome XR, roughly 15 million years ago (MYA). XR has acquired many properties typical of an X chromosome including complete MSL-mediated dosage compensation [20],[21], whereas the non-recombining homolog of this chromosome is almost entirely degenerated and heterochromatic [22], as expected of a Y chromosome.

Along the lineage leading to *D. miranda*, there has been an additional sex chromosome fusion [23] which arose ∼1 MYA [24], that of the Y chromosome to the autosome designated as chromosome 3 in *D. pseudoobscura* (Muller C, or 2R in *D. melanogaster*) [25]. This fusion formed a neo-Y that has now partially degenerated [25]–[29], with about half of the open reading frames (ORFs) of neo-Y genes disrupted by deletions, insertions, frameshift mutations, and premature stop codons [30]. Many neo-Y genes with these types of disruptions in ORFs likely fail to produce functional proteins, and many genes are transcriptionally silenced or expressed at a lower level from the neo-Y [30], and are thus truly hemizygous in males. At the same time, the neo-X of *D. miranda*, the formerly autosomal homolog of the neo-Y, is acquiring MSL-mediated dosage compensation [20],[31],[32], with about half of actively transcribed genes on the neo-X being targeted by the MSL complex [33].

## Results

### A Homologous Timecourse Of Expression Between Species

To take advantage of this snapshot into sex chromosome and dosage compensation evolution, we generated a timecourse of transcript levels in female and male embryos in *D. pseudoobscura* and *D. miranda*, using mRNA sequencing (mRNA-Seq).

We wanted to determine transcript levels during two important periods of embryonic development: the maternal to zygotic transition (MZT), when maternal transcripts begin to degrade and widespread zygotic transcription is initiated, and the onset of MSL-mediated dosage compensation (Figure 1B). Widespread activation of zygotic transcription occurs during stage 5, roughly 2.5 hours into development in *D. melanogaster*. The exact point at which MSL-mediated dosage compensation is established in *D. melanogaster* is unclear, but the histone modification that is deposited by the MSL complex (H4K16ac) is not detectable until stage 9 by antibody staining in *D. melanogaster* [13],[14]. To sample both of these important events, we began our timecourse at the earliest stage readily distinguished by morphological features (stage 2), sampled through mitotic cycles four and five, and then again before, during and after stage 9, for a total of eight timepoints.

Our single-embryo RNA sequencing approach was particularly beneficial here, as it allowed us to sample embryos from each species at precisely matched developmental stages based on morphology and not developmental time, which varies between species. It also allowed us to determine the sex of each embryo prior to sequencing and thereby select samples to generate sex-specific expression data.

We chose eight stages with readily identifiable diagnostic morphological features (Figure 1B). We collected, dechorionated, and imaged live embryos from each species, and harvested embryos at the desired stages, extracting both DNA and RNA for subsequent analysis. We used the DNA to determine the sex of each embryo using a PCR-based assay, employing redundant primer sets to detect the presence of a Y chromosome or two X chromosomes in *D. pseudoobscura*, and primer sets to determine the presence of a neo-X vs. neo-Y in *D. miranda* (see Methods). We chose three female and three male embryos for each species, for each of the eight stages, for a total of 96 samples (see Table S1). We prepared single embryo mRNA sequencing libraries from RNA from each of the selected embryos, without amplification of input RNA (see Methods), and sequenced the libraries on an Illumina HiSeq 2000 DNA sequencer.

### Data, And Distinguishing Maternal And Zygotic Transcripts

We aligned mRNA-Seq reads to the *D. pseudoobscura* reference sequence (Flybase release 2.25) or the *D. miranda* reference sequence [30] using Bowtie [34] and TopHat [35], and inferred transcript levels using Cufflinks [36]. We normalized transcript levels between samples so that the total inferred transcript levels of chromosomes 2 and 4 (which are autosomes in both species) were identical. Like in our previous study utilizing the single-embryo RNA-Seq technique, the data produced by this method are highly reproducible, with Spearman's rank correlation coefficients between replicate samples (same stage, same sex) exceeding 0.95.

In order to distinguish transcripts of zygotic origin from those deposited in the egg by the mother, we analyzed embryos that were all hybrids between genetically distinct parental lines within each species. For *D. pseudoobscura*, all mothers were Flagstaff 14 and all fathers PP1134. For *D. miranda*, all mothers were MSH22 and all fathers SP138. We used genomic sequence data for each pair of lines to identify 90,347 single nucleotide polymorphism (SNPs) fixed between the parental lines, and covered by transcripts in our dataset in *D. pseudoobscura* (92% of expressed genes in our dataset have SNPs). Similarly, in *D. miranda*, we identified 31,189 SNPs fixed between parental lines (75% of expressed genes in our dataset have SNPs), not counting those that distinguish the neo-X from the neo-Y (both *D. miranda* genomes were female, so additional information was needed to compare the neo-X to the neo-Y). This is consistent with a reduced level of polymorphism in *D. miranda* [37],[38]. We also identified 14,060 SNPs in coding regions of genes that distinguish the neo-X from the neo-Y, covering 68% of expressed genes on this chromosome, similar to the genome-wide average (though genes on the neo-X/neo-Y that have SNPs have more divergent sites than those elsewhere, due to the divergence between the neo-sex chromosomes).

For this study, we are primarily concerned with the zygotic transcript abundance for female and male embryos, as maternal mRNA deposition to female and male embryos does not differ. While mRNA-seq reads containing maternal alleles could be derived from either maternally deposited or zygotically transcribed RNAs, reads containing paternal alleles can only have come from zygotic transcription, and therefore can be used as a proxy for zygotic expression. We explored a number of different methods for defining which genes were zygotically expressed, all of which gave consistent results. Here, we use two definitions of zygotic expression for a gene. The first we primarily show (Figures 3, 4, and 6) is based on having a high proportion (∼50%, see Methods) of reads that are paternally derived; this is determined using female transcript levels (as males have no paternal X). The allele-specific definition of zygotic expression is used to analyze total transcript level. The second definition of zygotic genes is based on a gene having very low total expression at early stages and high total expression at later stages (Figure 5). This second definition does not depend on the allele-specific data, and so we use this definition for analysis of allele-specific transcript level (see Methods for further information and explanation of method choice, and also figure legends for descriptions of definitions used in each figure). Both methods call zygotic genes separately in each species, and for the allele-specific method, genes are called separately for each stage.

### Transcript Levels Are Largely Conserved Between The Species

**Figure 2:**
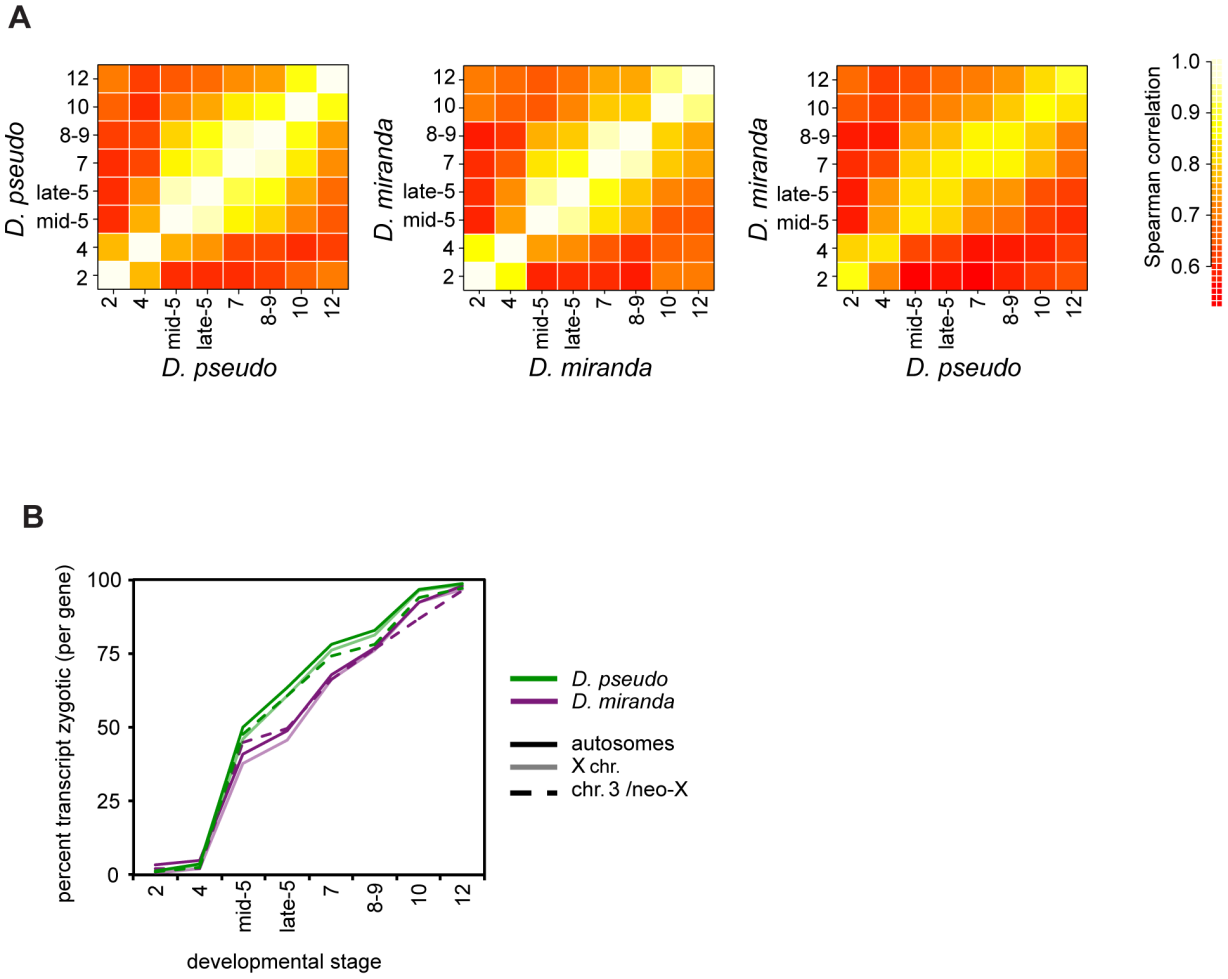
Timing of transcript abundances in *D. pseudoobscura* and *D. miranda*. A) A heatmap of pairwise correlation coefficients for mRNA abundance for genes (in RPKM) at all stages, averaged over all individuals per stage. The first two plots show within species comparisons, the third shows the between species comparison. *D. pseudoobscura* and *D. miranda* have very similar expression patterns at each stage, over this period of development. See Table S2 for numerical values. B) Percent of reads per gene that are zygotic in origin, estimated from the proportion of paternal reads (x2) over developmental time, for both species. These plots are derived from female embryos, as males have no paternal allele for X-linked genes. Solid lines are autosomes, pale lines are X chromosomes, and dotted lines the neo-XY/Chr.3. See Table S3 for significance tests of between-species differences.

Transcript levels across all genes for this period of early development are highly conserved between *D. pseudoobscura* and *D. miranda* (Figure 2A), across both females and males (the same stage between species, Spearman's rho of 0.85–0.92, as compared to a mean of 0.95 for replicates of the same stage within either *D. pseudoobscura* or *D. miranda*, see Table S2). This is as expected for closely related species, due to a high level of conservation of transcript level between morphologically homologous stages between the species, and supports the accuracy of our embryo staging based on morphology.

We used our allele-specific data to explore the activation of zygotic transcription in each species, using paternal reads as a proxy for zygotic transcription. Examining the average per gene proportion of zygotic reads (i.e. 2x fraction of allele specific reads that are paternal), we find that there is an offset in the timing of zygotic expression between the species (Figure 2B). *D. miranda* has significantly fewer zygotically derived transcripts per gene on average present from the beginning of zygotic transcription in stage 5, up to stage 8–9 or 10, where its zygotic transcript level equals *D. pseudoobscura* (see Table S3 for bootstrap confidence intervals). This pattern of lower levels of zygotic transcript per gene in *D. miranda* is species-specific, rather than chromosome-specific, as both autosomes and X chromosomes show the same fraction of zygotic transcripts within each species (Figure 2B).

Could the differences we observe in onset of zygotic transcription between *D. pseudoobscura* and *D. miranda* affect subsequent analyses? To address this, we first asked whether the same morphological stage in both species is also the best comparison based on transcript level. We examined pairwise correlations of transcript levels between both sexes and species, for both the set of all genes, and for autosomal genes (Figure S1 and Table S4). Individuals of a particular sex and stage in a species are always highly correlated with the same stage, regardless of sex. This holds across species as well, though the correlations are slightly lower. In the majority of cases, the highest correlation is between individuals of the same stage. Occasionally, a highly developmentally similar neighboring stage has a statistically slightly higher correlation, but this is not systematic across comparisons. Thus, we believe that there are no systematic differences in development rate that are unaccounted for by our sampling, either between species, or between sexes within a species. This would indicate that the transcriptional delay between these two species is less than a single stage by our sampling. Additionally, in subsequent analyses, we will define genes as zygotic separately for each species, and using the allele-specific zygotic definition, per stage. While we cannot rule it out, these factors should minimize the contribution of the delay in zygotic transcription in *D. miranda* to subsequent analyses.

### Onset Of Dosage Compensation In Development

**Figure 3:**
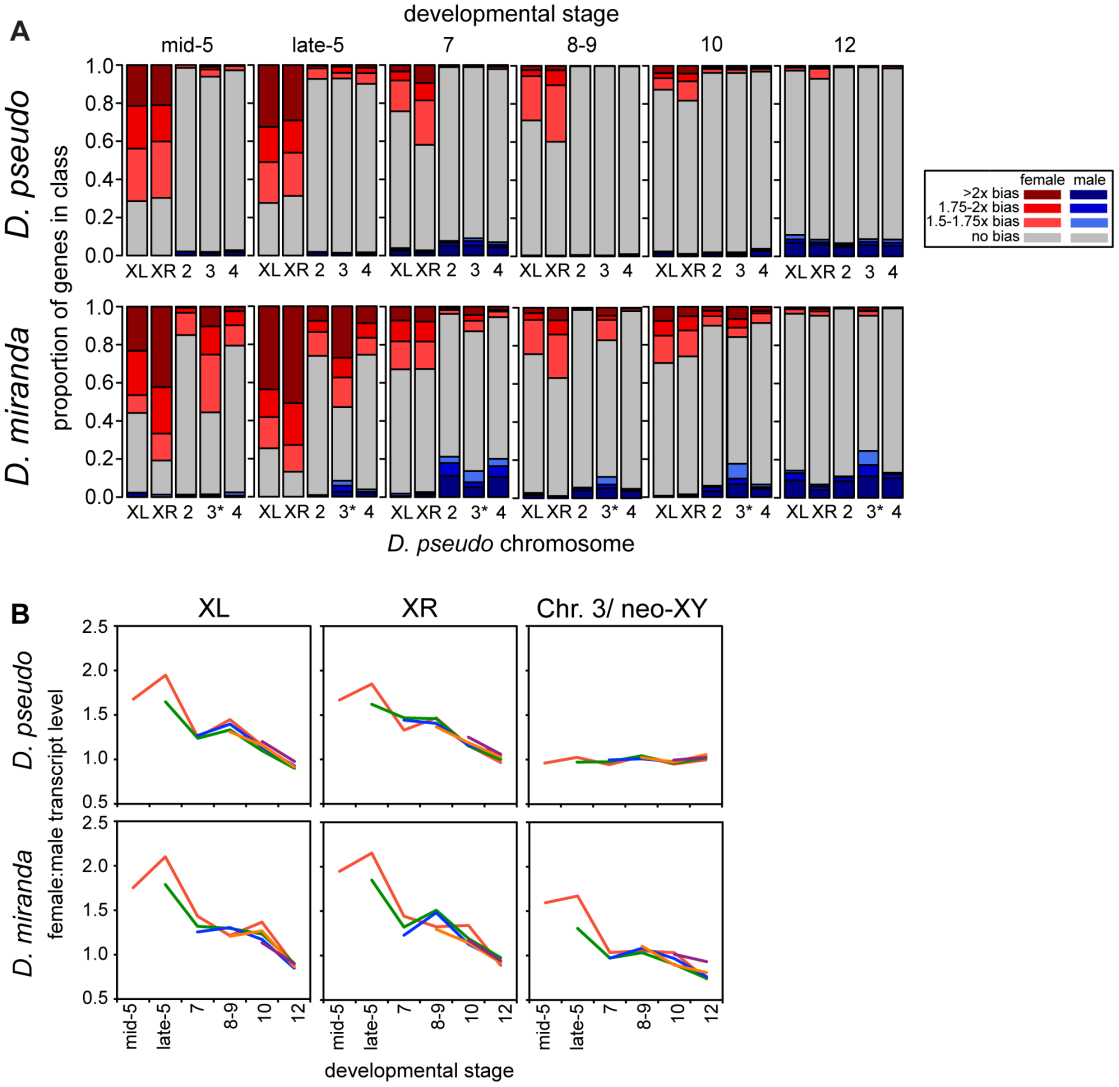
Onset of dosage compensation in *D. pseudoobscura* and *D. miranda*, on a genome-wide scale. A) Barcharts show proportions of zygotic genes in each sex-bias class, determined by the allele-specific zygotic definition, of female biased (shades of red), male biased (blue), and unbiased genes (grey), for each chromosome. *D. pseudoobscura* chromosome names were used, so the * is a reminder that this chromosome is the neo-XY in *D. miranda*, for this chromosome, reads from both the neo-X and neo-Y are included. As roughly half of the genes on the neo-Y are still producing transcript, this chromosome is currently a mix of genes that are hemizygous in males and those that have two functional copies, and perhaps unsurprisingly then, looks less female-biased than the ancestral X chromosomes. See Tables S5 and S7 for statistical analyses of the data in this figure. B) Female to male transcript ratio for genes grouped by when substantial zygotic transcription is first observed (see Methods for definition, lines are drawn originating in the stage where zygotic transcription is observed and indicated by color), show that the pattern of sex bias in expression is relatively constant within a stage, regardless of when genes become zygotically active.

At the onset of zygotic transcription, zygotic genes on the X chromosome are largely female biased. This female bias decreases over developmental time, and by stage 12, the X chromosomes are no more sex biased than the autosomes, indicating complete dosage compensation of zygotic genes (Figure 3A, Figure S2 for a different zygotic definition showing the same results). The onset of dosage compensation appears to be earlier in development in *D. pseudoobscura* than in *D. miranda*, though by stage 12, the two species reach equivalent levels of complete compensation (Figure 3A). The delay in onset of zygotic transcription in *D. miranda* may contribute to this, though we note that our zygotic genes are called in a stage and species-specific manner, minimizing the contribution of the delay to the compensation phenotype. As shown in Figure 3A, *D. miranda* has significantly more female-biased zygotic genes than *D. pseudoobscura* on XL and XR in nearly all developmental stages prior to stage 12 (See Table S5 and S6 for chi-squared test p-values for comparisons at various female-bias cutoffs). In early development, *D. miranda* has many zygotic genes on chromosomes XL and XR in the >2x female bias category, and significantly more than *D. pseudoobscura* until the last few stages, (Table S5). This is consistent with the view that the majority of zygotic genes in *D. miranda* are completely uncompensated at early stages, as then we would expect expression levels to be distributed reasonably symmetrically around 2x female bias.

Sex-biased expression is much less prevalent on the autosomes, but *D. miranda* does have a significantly higher female bias on the autosomes for the early stages of zygotic transcription (stage 5, see Tables S7 and S8). After these early stages, *D. miranda* autosomes lose their sex-bias, and if anything, become slightly male-biased. The increase in X-linked female bias might be promoting the significant female-bias on autosomes during the early developmental stages. For example, an uncompensated X-linked transcription factor could upregulate autosomal targets in females.

The gradual onset of dosage compensation could reflect the slow establishment of MSL-mediated dosage compensation chromosome-wide. Or, alternatively, it could be that the subset of genes expressed zygotically early in development are less likely to be dosage compensated throughout development. To examine this, we partitioned genes based on the stage at which they first meet our definition of being zygotically transcribed (see Methods). We then compared the extent of compensation of these different groups of genes at identical timepoints, and found that they had similar temporal patterns of dosage compensation (Figure 3B). This suggests that compensation status is a stage-specific, and not gene-specific property, and that by whatever mechanism compensation is occurring, the effect is genome wide. In addition to the mean behavior per gene, we visualized the sex bias for each gene (Figure S3) over development, which demonstrates the same overall pattern, where the female bias of early zygotic X-linked genes decreases in concert over developmental stages.

**Figure 4:**
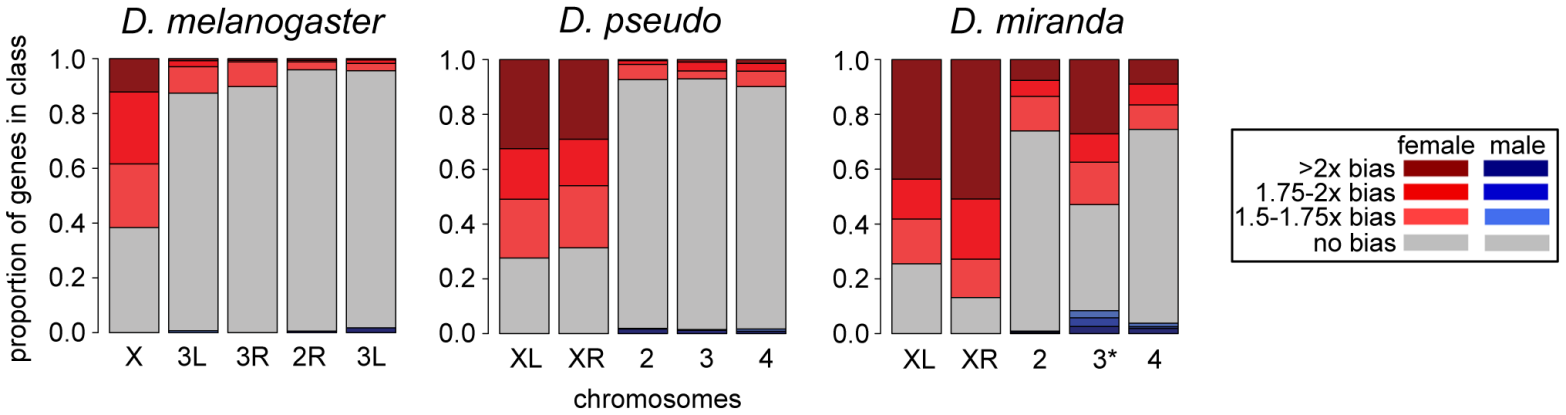
Sex-bias of transcripts in embryo at one timepoint across three species. Comparison of sex bias in transcripts for zygotically transcribed genes (using the allele-specific zygotic definition) in *D. pseudoobscura*, *D. miranda*, and *D. melanogaster*, at one stage (late stage 5, or the end of blastoderm) early on in development, before the activation of MSL-mediated dosage compensation. Chromosomes are in the same order for all species, *D. melanogaster* has only the ancestral X, *D. pseudoobscura* and *D. miranda* both have XL and XR, and *D. miranda* additionally has the neo-XY chromosome (indicated with 3*). The neo-XY levels shown include those from both the neo-X and the neo-Y in males, and as many of these genes on the neo-Y are still producing transcript in males, this chromosome appears to have less female bias than the ancestral X chromosomes.

How does this compare to our previous results for the pre-MSL early-zygotic period in *D. melanogaster*? In *D. melanogaster* roughly 20% of the genome is in sex chromosomes, in *D. pseudoobscura* it is 40% and 60% in *D. miranda* (though roughly half of the genes on the neo-X/Y are not truly hemizygous in males, as the neo-Y copies are likely still functional). When we compared the same developmental stage (late Stage 5) between *D. miranda*, *D. pseudoobscura* and *D. melanogaster* (using our earlier data described in [15]), *D. melanogaster* has the least female bias, and *D. miranda* the most (Figure 4, Figure S4 for different zygotic definition showing similar results). Comparing just the homologous X chromosome (X in *D. melanogaster*, XL in the other species), *D. melanogaster* is significantly less female-biased than either *D. pseudoobscura* or *D. miranda* (p<0.002, Wilcoxon rank sum test). The comparison between *D. pseudoobscura* and *D. miranda* for this same chromosome and stage is borderline significant (p = 0.05, Wilcoxon rank sum test), with *D. miranda* showing more female bias. The relatively low level of significance for this *D. pseudoobscura* and *D. miranda* comparison may be due to the relatively small number of genes on XL, as the XR comparison between *D. pseudoobscura* and *D. miranda* is significantly different (p = 2×10^−8^, Wilcoxon rank sum test) and the magnitudes of the differences in median are similar for XL and XR (1.7∶1.9x female bias for XL; 1.7∶2x for XR). As the X we are comparing between all three species is the ancestral X chromosome of all *Drosophila* species, the lack of dosage compensation in *D. pseudoobscura* and *D. miranda* is not simply a product of insufficient time to evolve dosage compensation.

**Figure 5:**
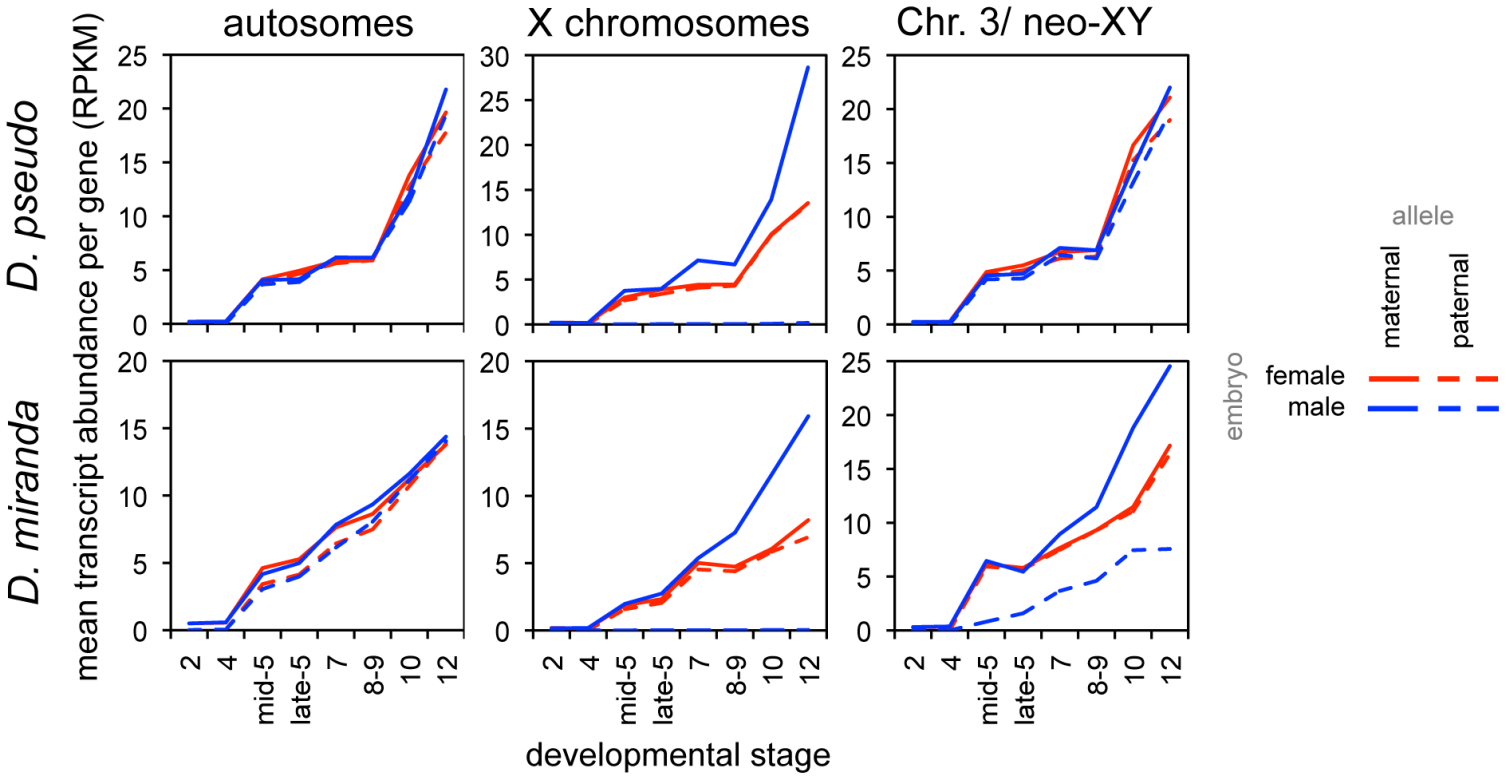
Onset of dosage compensation for zygotically transcribed genes, as revealed by allele-specific expression. Mean proportion of reads per gene attributable to the maternal and paternal alleles, in both female and male embryos, for zygotic genes (defined by transcript level, see Methods). While there are no significant differences in transcript level from the autosomes, the single male X begins to be transcribed at a higher level than either female X, resulting in compensation of chromosome dosage. Unlike *D. melanogaster* [15], these two species show several stages of zygotic expression with the male X having the same transcript abundance as the female Xs, indicating a period with, on average, no dosage compensation (the *D. melanogaster* study was of a tighter distribution of stages, and ended at late stage 5).

To further explore the dynamics of dosage compensation onset, we examined transcript levels of zygotic genes (defined based on total expression levels, see Methods) in an allele-specific manner. In the early stages of expression for zygotic genes, during stage 5, we observe that both the single male X and each of the two female X chromosomes have similar average transcript levels, indicating no dosage compensation for these stages, in contrast to what we have previously found in *D. melanogaster* [15]. While *D. melanogaster* showed some dosage compensation from the beginning of zygotic transcription, the average zygotic gene in *D. pseudoobscura* and *D. miranda* has no dosage compensation for several stages after the onset of zygotic transcription. The allele-specific transcript abundances show the onset of dosage compensation, as the single male X chromosome begins to have significantly more zygotic transcripts per gene than each of the two female X chromosomes (Figure 5, Table S9; also see Figure S5, Table S10 for a different zygotic definition). By the end of this period of development, the male X is transcribed at twice the level of each female X in both species, indicating that dosage compensation has been fully established. This reduction in female bias over time is consistent with the predicted onset of MSL-mediated dosage compensation. We are likely observing the onset of zygotic transcription without dosage compensation followed by the onset of MSL-mediated dosage compensation in these species.

There are differences in the timing of onset of dosage compensation in the two species. For an average zygotic gene, the single maternally derived X chromosome in males has a significantly higher transcript level than the maternal X in females (with the mean level for males falling outside the 95% bootstrap confidence interval for the female mean for both XL and XR) at stage 7 in *D. pseudoobscura*, whereas this is observed later in *D. miranda*, between stages 8 and 9 (Table S9, S10). Again, the delay in onset of zygotic transcription in *D. miranda* may contribute to this result, though we do not believe that the delay in zygotic transcription creates an offset equivalent to an entire stage between the species (see above). There are no significant differences between transcript abundances of autosomes by this same method.

### Comparing Embryonic Dosage Compensation To Msl Binding Data

We had hoped to use our expression data and chromatin immunoprecipitation sequencing (ChIP-seq) data for MSL binding in *D. miranda* larvae [33], to observe the process of MSL-mediated dosage compensation being established in physical space along the chromosome. The MSL complex is thought to bind initially at high affinity or chromatin entry sites (HAS or CES; [39],[40]), and subsequently spread along the chromosome [10]. We first asked if compensated or uncompensated genes were closer together than we would expect by chance. There was no detectable signal of this, when we took into account that zygotic genes are themselves closer than expected (see Methods). We then examined genes based on their proximity to predicted HAS in *D. miranda* [33], and found that genes that were closer to HAS were no more likely to be compensated than those further away (see Methods), at any stage. We also examined the average H4K16ac enrichment per gene from ChIP-seq data in *D. miranda* larvae [33], and found that by stage 12, genes that were dosage compensated in the embryo were significantly more likely to be enriched for H4K16ac than non-compensated genes (with both sets falling outside the 95% bootstrap confidence intervals of the other category, see Table S11). This is consistent with genes compensated by stage 12 in our embryo data being targets of the MSL complex. Interestingly, at the earliest stage of widespread zygotic expression, mid-stage 5, we find that genes that are compensated in our data are significantly less likely to be enriched for H4K16ac in larvae than uncompensated genes (see Table S11, for bootstrap confidence intervals). This suggests that the small numbers of compensated genes found during this period are not compensated later by MSL, or are not being transcribed in the larval stage, and perhaps then have distinct compensation mechanisms. While we are able to observe the effects on gene expression before or after the onset of MSL-mediated dosage compensation, we are unable with this data to observe the process of establishing the MSL compensation mechanism (e.g. we do not find that genes that are compensated early during the establishment of MSL are nearer HAS sites). Perhaps we do not have appropriate time resolution of samples during this time period, or perhaps the genes targeted by MSL are largely different in embryos than they are in larvae. More mechanistic studies of the onset of MSL-mediated dosage compensation in a developmental context are needed.

### Dosage Compensation And The Age Of The X Chromosome

In examining zygotic X chromosomal gene expression in *D. pseudoobscura* and *D. miranda*, we can compare X chromosomes of three evolutionary ages: XL, the X ancestral to all *Drosophila* species; XR, which arose ∼15 MYA; and the neo-X in *D. miranda*, which arose 1–1.5 MYA.

Chromosome XR has been shown in previous studies to possess all the properties of an X chromosome, and in many ways it is indistinguishable from the ancestral X [20],[21],[33]. Our findings for transcript levels for both female and male embryos for the developmental periods both prior to and during the onset of MSL-mediated dosage compensation support this conclusion.

The dosage compensation status of the neo-X chromosome is more complicated to assess, as the neo-Y chromosome still possesses functional copies of many genes (about half of the genes, [30]). When we combine reads from the neo-X and neo-Y (which we refer to as neo-XY) to count total transcripts in males for each gene, we find that it is better compensated at early stages than either of the older X chromosomes (Figures 3A, 3B, 4). This result is potentially due to retained transcription of neo-Y copies of genes.

To distinguish whether the compensated genes on neo-XY are due to neo-Y transcription or due to a better-compensated neo-X, we again examined our allele-specific data to distinguish neo-Y reads from neo-X reads (see Methods). We find generally lower levels of transcripts from the neo-Y (Figure 5, dashed blue line) compared to the neo-X (Figure 5, solid blue line). Additionally, there is a striking pattern of the single neo-X in the male showing a higher mean transcript level than either neo-X in the female (Figure 5). The higher transcript level from the single male neo-X is similar in its temporal pattern to the compensation we see on the evolutionarily older X chromosomes. Thus, consistent with studies in larvae and adults [20],[31]–[33], the neo-X in *D. miranda* is to some degree dosage compensated at later stages, presumably through the onset of MSL-mediated compensation.

**Figure 6:**
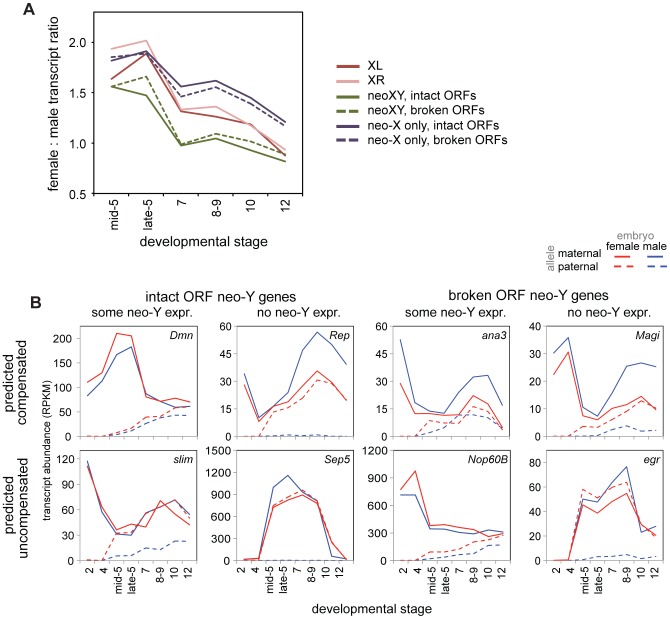
The neo-XY of *D. miranda* and the evolution of dosage compensation in the embryo. A) Female to male transcript ratios over stages for zygotic genes (allele-specific definition, see Methods) on the neo-XY (with transcripts from both the neo-X and neo-Y included), and for just the neo-X, both broken down by whether the neo-Y copy of that gene is predicted to be functional (intact ORF) or non-functional (broken ORF). See table S12 for Wilcoxon p-values comparing chromosomes over stages. B) Example genes from the neo-XY, with allele-specific transcript abundances. Every combination of predicted functionality of the neo-Y (with the assumption that genes with intact ORFs are functional, genes with broken ORFs are nonfunctional), contribution of neo-Y transcript, and predicted compensation status (relative to the predicted functionality of the neo-Y copy of the gene) is observable.

When determining compensation status of a gene on the neo-XY, we must consider whether the neo-Y copy of the gene is functional. Transcripts are often produced whether the gene is capable of making a functional protein or not, in some cases then, total transcript level is not an accurate measure of functional dosage compensation. A previous study [30] classified genes on the neo-Y as having an intact or disrupted open reading frame (ORF). The ORF disruptions are due to frameshift mutations, premature stop codons, or sizable deletions, and thus make the neo-Y copy of the gene unlikely to make a functional protein. It is these genes that are likely to be, on the functional level, truly hemizygous in males, and thus in need of compensation. When we ask specifically about compensation in genes with or without intact ORFs, we find that both are equally well compensated, both early and late in embryogenesis (Figure 6A, Wilcoxon test p-values in Table S12). Thus, dosage compensation of any gene on the *D. miranda* neo-X in the embryo is generally uncorrelated with the neo-Y status of that gene (Figure 6A). This observation is consistent with findings from more mechanistic studies of the degradation and down-regulation of the neo-Y and acquisition of MSL dosage compensation on the neo-X, where the two processes appear to be independent from one another and primarily driven by the ancestral background chromatin state of the neo-sex chromosome [41].

This is apparent on a gene-by-gene level as well, for we observe genes with all combinations of predicted functionality, contribution of neo-Y transcription, and compensation status (Figure 6B). Were degradation of the neo-Y coordinated with the acquisition of dosage compensation, we would expect an overrepresentation of genes with disrupted ORFs amongst the compensated genes. Instead, we observe genes with disrupted ORFs who have not acquired dosage compensation, genes with intact ORFs who retain neo-Y transcripts who are precociously dosage compensated (as genes with functional neo-Y copies are not in need of dosage compensation), as well as every other combination (Figure 6B).

Ultimately, to investigate how hemizygous genes in males on the neo-X are compensated in *D. miranda*, we must examine only neo-X transcript levels from genes with non-functional neo-Y ORFs. When we examine transcript levels from just the neo-X over developmental time (Figure 6A), we observe a significant difference between the neo-X and the two older X chromosomes (Wilcoxon test, almost all comparisons significant at p<0.05, see Table S12). In the earliest stages of zygotic transcription, XL, XR, and the neo-X are similarly female biased in *D. miranda*; as early as stage 7, female bias begins to decrease in XL and XR relative to the neo-X. Through later stages, the neo-X decreases along a similar trajectory to XL and XR, but is always ∼0.25x more female biased. This suggests that early zygotic expression is similarly compensated (i.e. a lack of compensation) between all of the *D. miranda* X chromosomes, but that the *D. miranda* neo-X has less effective (presumably MSL-mediated) dosage compensation at later stages. This is consistent with findings in larvae, where only about half as many genes are dosage compensated by MSL on the neo-X as compared to XL or XR [33].

## Discussion

In this study, we examine sex-specific transcript abundance across embryonic development, both before and after the onset of MSL-mediated dosage compensation. We find significant differences between species in the period prior to the onset of MSL-mediated compensation, where we had previously demonstrated in *D. melanogaster* that roughly half of zygotically expressed genes on the X chromosome are fully compensated. Conversely, for *D. pseudoobscura* and *D. miranda*, we find a general lack of compensation for X chromosomal genes during this pre-MSL period. We have also provided evidence for a delay in the onset of the later, more effective, MSL-mediated dosage compensation in *D. miranda* relative to *D. pseudoobscura*.

The difference we have demonstrated in the effectiveness of compensation during the period of early zygotic expression is striking. X-linked genes in *D. melanogaster* genes have, on average, some degree of dosage compensation from the very beginning of zygotic expression, with the single male X having a higher transcript abundance than either single female X [15]. In contrast, genes in *D. pseudoobscura* and *D. miranda* male embryos have on average no dosage compensation until after gastrulation (or later in *D. miranda*), as the single male X has an average transcript level that is exactly the same as either one of the two female Xs, indicating that transcript level is entirely proportional to gene copy number (Figure 5). This indicates that either *D. pseudoobscura* and *D. miranda* lack a mechanism or some set of mechanisms that *D. melanogaster* possesses, or that the mechanism(s) are overwhelmed by the number of X-linked genes in the former two species.

The average level of early zygotic dosage compensation we observe in *D. melanogaster* embryos [15] is consistent with studies on changes in dosage of autosomal genes [42]. In flies heterozygous for autosomal deficiencies of varying size, the mean transcript level of single copy genes is approximately 1.5x (where 2x is the expression level of two copy autosomal genes), although there is considerable variance around this level for individual genes [43]–[46]. In *D. melanogaster*, we also found the female to male ratio for genes on the X chromosome for the early zygotic stages (Stage 5, mitotic cycle 14) was on average, 1.5x [15]. This raises the possibility that both early zygotic X and autosomal aneuploidy dosage compensation might be due to a general mechanism that recognizes aneuploid genomic regions in *D. melanogaster* and compensates the expression of included genes, or that homeostatic mechanisms in some or all gene networks respond to abnormal levels of transcription by altering regulation of parts of the network [44],[46].

However, in this study, we observe an average female to male ratio of 1.8x in *D. pseudoobscura* and nearly 2x in *D. miranda* for genes on XL and XR at these developmental stages, which is not consistent with the autosomal level of compensation for differences in gene dose observed in *D. melanogaster*. It is then possible that these species vary in their capacity to compensate for dosage differences in all stages or chromosomes, which could be tested by doing similar autosomal dose variation studies in species of the *D. pseudoobscura* lineage to those cited above for *D. melanogaster*. Or, it may be that the mechanism(s) responsible for X dosage compensation at the early zygotic stage of development is overwhelmed by the much larger number of genes that are present in a single copy in males of these species with many more X-linked genes.

There is precedence for a limit of the number of genes that can be dosage compensated, also from the autosomal studies in *D. melanogaster*. In a classic 1972 paper, Lindsley et al. showed that *D. melanogaster* can tolerate single-copy deletions of roughly 1% of the genome (1.5 Mb), and 3% is usually lethal [47]. The deficiencies described by [44],[48] are smaller than 1 Mb, and thus less than 1% of the genome. Additional studies have suggested that with increasing size of deletion, the additive effects of changing the dosage of an increasing number of individual genes results in the collapse of gene networks [44],[49], which could be responsible for the lethality in these animals. The X chromosome, at roughly 1/5 of the genome in *D. melanogaster* already exceeds the 3% viability threshold suggesting that the mechanism(s) conferring autosomal dosage compensation may not be the source of early zygotic dosage compensation in *D. melanogaster*. Additionally, these studies show that mechanism(s) that compensate for difference in gene dosage may have difficulty when the number of genes that are hemizygous increase, perhaps as with our species with larger numbers of X-linked genes.

We have observed both a difference in early zygotic dosage compensation, and potentially a difference in timing of onset of MSL-mediated dosage compensation. Both findings are consistent with dosage compensatory mechanisms being overwhelmed in species where a larger percentage of the genome is held in sex chromosomes. Or, these findings may be explained by a lineage specific gain or loss of early zygotic dosage compensation mechanism(s). Additional studies of both the early zygotic stages and the onset of MSL-mediated compensation, with additional species of varying karyotype, are needed to distinguish between these possibilities. With transcript profiles of these stages from a number of additional species, we should be able to determine if there is a correlation between the effectiveness of compensation and the proportion of the genome held in sex chromosomes, or alternatively identify the point on the Drosophila phylogeny when the early zygotic dosage compensation phenotype was either gained or lost.

Whatever the mechanistic basis of the difference in dosage compensation, additional studies are also needed to address the next obvious question: are there consequences of these differences in effectiveness of early zygotic dosage compensation and timing of the onset of MSL-mediated compensation? One could imagine several different outcomes. It could be that these (and other) species with less effective early dosage compensation or later onset of MSL-mediated dosage compensation will result in sex-specific phenotypic effects, and greater degrees of sexual dimorphism. Or, the inherent robustness of developmental systems will result in sex-specific differences in transcription having limited or no phenotypic effect on some or many developmental phenotypes. While many developmental processes have steps that are concentration-dependent, for example the number of transcription factor molecules in a particular space, it is exactly these kinds of processes that have been demonstrated to be the most robust to genetic, environmental, and stochastic variation. Understanding the developmental consequences of the variation in sex chromosome dosage will be critical to understanding how different mechanisms of dosage compensation evolve.

## Methods

### Fly Lines, Imaging, And Sample Acquisition

Flies were raised on standard media at 18°C (preferred temperature of these species). Virgin females of the *D. miranda* MSH22 line were crossed to *D. miranda* SP138 males, and *D. pseudoobscura* Flagstaff 14 virgin females were crossed to *D. pseudoobscura* PP1134 males. Eggs were collected from these crosses, with mothers being between 5–10 days old, and numerous enough (hundreds) to minimize chances of multiple sample embryos coming from a single mother. After collection, eggs were dechorionated, placed on a slide in halocarbon oil, and visualized using light microscopy with a DIC filter on a Nikon Eclipse 80i microscope, with a Nikon DS-UI camera, and the NIS Elements F 2.20 software. Morphological features were used to collect embryos from stages 2, 4, mid stage 5, late stage 5, stage 7, the threshold between stages 8 and 9, stage 10, and stage 12, for a total of 8 stages. Embryos were then moved from the slide, cleaned from excess oil, and placed in a drop of TRIzol reagent (Invitrogen) within a minute or less of imaging. Embryos were then ruptured with a needle, allowed to dissolve, and moved to a tube with more TRIzol reagent, which was then frozen at −80C until extraction.

### Rna Extraction, Genotyping, Sequence Library Preparation

RNA and DNA extraction from single embryos was done with TRIzol (Invitrogen) reagent according to manufacturers protocol, except with a higher volume of reagent relative to the amount of material (i.e. starting with 1 mL of TRIzol despite expecting very small amounts of DNA and RNA). Extracted DNA was amplified using the Illustra GenomiPhi V2 DNA Amplification Kit (GE Healthcare), and genotyping for sex was performed with PCR. *D. pseudoobscura* samples were genotyped by using Y specific primers described in Carvalho and Clark, 2005 [22], as well as from primers developed to distinguish X chromosomes in the two *D. pseudoobscura* lines (Flagstaff 14 and PP1134): pse_XX_L TGCTAAACATAATCAAGAGCGGCATCA pse_XX_R TTCCCAAGCTAGACGGACAACAGAAAC. For the *D. miranda* lines, we used two different sets of primers designed to distinguish the neo-X from the neo-Y, (mir_neoXY_1_L TGACTTACTGCTCACCATTGGCACACT, mir_neoXY_1_R TTGCTGGAATGAGTACGCATAGCCTCT; mir_neoXY_2_L GACCAGCATAAGTTCCCAAGGAGGAGT, mir_neoXY_2_R AATGACTGGGCAACTGTTTAACTTCA. From each species, 3 females and males were chosen for each of the 8 stages, resulting in 48 samples. The genotyping assay was imperfect, resulting in a few stages with differing numbers of males and females, the samples and their properties are listed in table S1. Extracted RNA was treated with TurboDNase (Ambion), mRNA-Seq library preparation was performed with Illumina TruSeq RNA kits, and samples were indexed to run 12 samples per sequencing lane, and sequenced on an Illumina HiSeq sequencer.

### Data, Snp Calling, Allele-Specific Rna-Seq

Reads from each RNA-Seq sample were mapped to either the *D. pseudoobscura* genome (Flybase release 2.25) or the *D. miranda* genome (http://www.ncbi.nlm.nih.gov/genome/10915), using Bowtie and Tophat, and transcript abundances for annotated RNAs were called by Cufflinks. Data from each sample were normalized so that the total expression (reads per kb of sequence, per million mapped reads; RPKM) of autosomal genes (chromosomes 2 and 4) was constant. Analysis was also performed with normalization in *D. pseudoobscura* to all the autosomes in this species (chromosomes 2, 3, and 4), as well as normalization to total read number rather than autosomal levels, all the normalization procedures we explored produced the same results relative to the X and neo-X chromosomes.

SNPs were called from genomic DNA in both *D. pseudoobscura* lines and both *D. miranda* lines by aligning reads using Bowtie 2 (2.0-beta5, [50]), and using both Samtools (0.1.18, [51]) and GATK's Unified Genotyper [52],[53], and combining the SNPs called by these different methods using GATK. Called SNPs were kept only in the case where they were homozygous within both lines and differed between lines. Parameters were generally set to be permissive, as only SNPs that were covered and validated by RNA-Seq reads were used for allele-specific RNA-Seq analysis. SNPs between the neo-X and neo-Y chromosomes in *D. miranda* were obtained from aligning reads between neo-X and neo-Y with SOAPaligner (v2.21), and calling SNPs with SOAPsnp (v1.03), the SNPs were further filtered by the number of reads (>3 unique reads) supporting their existence. Methods for determining the predicted functionality of the neo-Y genes are detailed in [30]. To examine the percentage of genes with SNPs in our dataset, since only SNPs covered by reads were used, we first calculated the proportion of genes with at least one read (referred to in the Results as expressed genes), and determined the proportion of genes with SNPs compared to this total number of expressed genes.

We identified all RNA-Seq reads overlapping SNPs that varied between strains, and counted the frequencies of reads that can be attributed to the maternal or paternal allele (using a custom python script). All maternal or paternal reads for each SNP per gene are combined, and divided by the number of SNPs per gene.

All sequencing reads and processed data files used for analysis have been deposited in NCBI/GEO under accession number GSE53483 (http://www.ncbi.nlm.nih.gov/geo/query/acc.cgi?acc=GSE53483).

### Determining Zygotic Transcripts, Female To Male Transcript Ratios

All analysis was performed in R (version 2.15.2, [54]). We used two definitions of zygotic genes, each determined for each species separately. One was based on the allele specific data (allele-specific definition), and determined which genes were zygotically expressed on a stage by stage basis, this required that the gene be above a minimum read level (>5 RPKM) and the paternal allele was greater than 40% of total transcript level (maternal+paternal reads), this is used in Figures 3, 4 and 6. As males only had one allele for X chromosomal genes, we used the females to determine zygotic genes. We also used (but only show here in Figure 5) a definition of zygotic genes (transcript level definition) that simply required the gene to not have any maternal deposition (<5 RPKM at stage 2) and have read numbers >5 later in subsequent stages. Both of these methods had advantages, the allele specific definition is stage and species specific, and allowed for more genes to be counted as zygotic in later stages, as genes with maternally deposited mRNAs transitioned to zygotic transcription. The transcript level method allowed for more genes to be counted as it was based on total transcript level, and not necessary to have allele-specific data for that gene. We chose to use the transcription zygotic definition for Figure 5, because it avoids the circularity of conditioning an allele specific result on an allele-specific definition of zygotic activity. For Figure 2B, the percent of zygotic transcript was determined from the mean proportion of transcript attributable to the maternal and paternal allele per gene, depicted levels come from female embryos (due to the paternal allele on the X chromosome not being present in males). We determined the onset of transcription for individual genes (Figure 3B) by identifying the first stage they met our stage-specific definition of zygotic transcription. Female to male ratios of transcript abundance were determined for each developmental stage for those genes whose means across both females and males were above 5 RPKM. Figures 3, 4 and 6, show female to male ratios for various chromosomes, groups of genes, stages, and species, for allele-specifically determined zygotic genes. Keeping these definitions constant produced a slightly different graph for *D. melanogaster* in Figure 4 than previously published [15].

### Compensation In Space

To understand whether compensated genes were physically clustered on chromosomes, we calculated the mean distance from each compensated gene to the nearest compensated gene, and from each uncompensated gene to the nearest uncompensated gene. We compared these distances to a null distribution where we resampled zygotic genes at each timepoint, and similarly calculated the mean distance to the nearest compensated or uncompensated gene for each of the null replicates. Compensated or uncompensated genes are not on average closer together than what we would expect from a random sample of zygotic genes at any timepoint. It was necessary to draw the null distribution from zygotic genes, as zygotic genes are consistently closer together than expected by chance (compared to a null distribution resampled from all genes at each timepoint), this is observed on all chromosomes and across most stages.

Using MSL high affinity binding site (HAS) data reported in [33], we tested for an association between compensation status and physical proximity to MSL high affinity sites. Over all chromosomes, we calculated the distance of each gene to nearest MSL HAS, and looked at mean sex ratio in lower quartile of the distance to the nearest MSL HAS compared to the upper three quartiles. The mean sex ratio in the lower quartile of the distributions of distances to the nearest MSL entry site looked no more compensated than the upper three quartiles (Wilcoxon test), in any stage.

We partitioned our data into compensated and uncompensated and calculate the mean H4K16ac enrichment (data reported in [33]) over genes in each of these categories. In the earliest stage with zygotic expression, compensated genes on XL and the neo-X were significantly depauperate of H4K16ac binding, XR was not significant, possibly due to low sample size. In contrast, compensated genes at the last stage (stage 12) on, XL, XR, and the neo-X were marginally significantly associated with high H4K16ac binding. Thus, compensated genes in the early zygotic expression stages are not the same genes that are compensated by MSL in the larvae, and the genes compensated later in this period of development are targets for MSL-mediated compensation.

## Supporting Information

Figure S1 Heatmap of pairwise correlation coefficients between sexes, for mRNA abundance for all genes (in RPKM) at all stages, averaged over all female or male individuals per stage within a species. Both within and between species comparisons are shown. A) shows these plots based on autosomal genes, B) shows these comparisons based on all genes. Statistical analysis shown in Table S4.
(PDF)

Figure S2 Onset of dosage compensation in *D. pseudoobscura* and *D. miranda*, on a genome-wide scale (Figure 3 with an alternate zygotic gene definition). Zygotic definitions were determined in each species as in Figure 3, but genes used were only those classified as zygotic in both species at each stage. Barcharts show proportions of zygotic genes in each sex-bias class, determined by the allele-specific zygotic definition, of female biased (shades of red), male biased (blue), and unbiased genes (grey), for each chromosome. *D. pseudoobscura* chromosome names were used, so the * is a reminder that this chromosome is the neo-XY in *D. miranda*, for this chromosome, reads from both the neo-X and neo-Y are included. As roughly half of the genes on the neo-Y are still producing transcript, this chromosome is currently a mix of genes that are hemizygous in males and those that have two functional copies, and perhaps unsurprisingly then, looks better compensated than the ancestral X chromosomes. See Tables S6 and S8 for statistical analyses of the data in this figure.
(PDF)

Figure S3 Female to male ratio over stages for all zygotic genes at a particular stage. For a species, the female to male ratio of the zygotic genes at either mid-stage 5 or stage 12 are plotted for the rest of the stages. The color is based on the quantile of the female to male ratio at the stage conditioned on, at the top of the column, with the most female-biased genes in red, least female-biased genes in pale yellow. Female biased genes at early stages (red) are more likely to be less female biased at later stages (yellow), and vice versa, an effect observed most strongly on the autosomes.
(PDF)

Figure S4 Sex-bias of transcripts in embryo at one timepoint across three species (as in Figure 4) using two alternative zygotic definitions (A & B). Comparison of sex bias in transcripts for zygotically transcribed genes (using the allele-specific zygotic definition) in *D. pseudoobscura*, *D. miranda*, and *D. melanogaster*, at one stage (late stage 5, or the end of blastoderm) early on in development, before the activation of MSL-mediated dosage compensation. Chromosomes are in the same order for all species, *D. melanogaster* has only the ancestral X, *D. pseudoobscura* and *D. miranda* both have XL and XR, and *D. miranda* additionally has the neo-XY chromosome (indicated with 3*). The neo-XY levels include transcript levels from both the neo-X and the neo-Y in males, and as many of these genes on the neo-Y are still producing transcript in males, this chromosome appears to have less female bias than the ancestral X chromosomes. A) Zygotic definitions were determined in each species as in Figure 4, but the genes used were only those classified as zygotic in both *D. pseudoobscura* and *D. miranda*. B) Sex-bias of genes defined as zygotic in *D. melanogaster* (as in Figure 4), plotted in all species. Using this definition (B), *D. melanogaster* appears to have similar patterns of sex-bias as in A and Figure 4, whereas *D. pseudoobscura* and *D. miranda* have fewer female-biased genes, but still more highly female-biased genes than *D. melanogaster*. We note in both of these comparisons, genes determined to be zygotically transcribed in one species at this timepoint are not necessarily zygotically transcribed in the other species. Also the numbers of genes are greatly reduced using these gene lists, so results are more likely to be noisy. We were unable to use a shared zygotic gene list across all species using allele-specific zygotic definitions, as this reduced the gene number to just a handful of genes on each chromosome.
(PDF)

Figure S5 Onset of dosage compensation for zygotically transcribed genes, as revealed by allele-specific expression (as in Figure 5) with alternate zygotic gene definition. Zygotic genes were determined in the same manner for each species as in Figure 5, but only genes that were categorized as zygotic in both species were used in this Figure. Mean proportion of reads per gene attributable to the maternal and paternal alleles, in both female and male embryos, for zygotic genes (defined by transcript level, see Methods). While there are no significant differences in transcript level from the autosomes, the single male X begins to be transcribed at a higher level than either female X, resulting in compensation of chromosome dosage. Unlike *D. melanogaster* [15], these two species show several stages of zygotic expression with the male X having the same transcript abundance as the female Xs, indicating a period with, on average, no dosage compensation (the *D. melanogaster* study was of a tighter distribution of stages, and ended at late stage 5).
(PDF)

Table S1 Description of samples used. This table provides information on the 96 embryos sequenced for this study: their predicted sex from the pre-sequencing genotyping, their actual sex as determined by sex-specific gene expression patterns and the allele-specific data, which lane they were sequenced in, the Illumina index adapter number, and any notes on that particular sample.
(XLSX)

Table S2 Correlation coefficients within and between species. Table of pairwise Spearman's correlation coefficients for mRNA abundance for genes (in RPKM) at all stages, averaged over all individuals per stage. The first two tables show within species comparisons, the third shows the between species comparison. *D. pseudoobscura* and *D. miranda* have very similar expression patterns at each stage, over this period of development. These are the values displayed in the heatmaps of Figure 2A.
(XLSX)

Table S3 Significance of zygotic genome activation offset between *D. pseudoobscura* and *D. miranda*. Here we show the mean fraction of reads per gene that are zygotic in origin, estimated by doubling the paternal read fraction. We used female embryos, so that we might also count paternal reads for the X chromosomes. 95% bootstrap confidence intervals were calculated for the mean by resampling genes with replacement, taking the mean, and finding the 2.5% and 97.5% quantiles over bootstrap simulations. Values in red are those that fall outside the 95% confidence intervals of the other species.
(XLSX)

Table S4 Pairwise correlation coefficients by stage, sex, and species. Top table for each set is the pairwise Spearman's rank correlation coefficient between each stage in each comparison. The bottom set lists the observed pairwise correlation at each stage, the 95% bootstrap confidence intervals, and the maximum p-value for each column and row is reported. The bootstrap confidence intervals were constructed on the observed correlation corresponding to the diagonal (same stage in both comparisons. This was constructed by resampling genes with the replacement and recalculating the correlation many times, the 95% confidence interval was constructed from these resampling simulations. The row p-value shows the p-value of whether the maximum correlation of a row is significantly higher than the correlation found on the diagonal, i.e. it shows whether another stage is significantly better correlated than the homologous stage. These p-values were constructed as in the confidence interval bootstrap simulations. Yellow indicates the homologous stages between the two timecourses compared in that set, orange values are those where a non-homologous stage has a significantly higher correlation, and red indicates the significant p-values.
(XLSX)

Table S5 Comparing female-biased genes between *D. pseudoobscura* and *D. miranda* across stages. We show a range of different cutoffs of female bias, from >1.5x–>2x compared to male level. A chi-squared p-value is shown for the proportion of genes falling above the cutoff between species. Significant p-values are highlighted in yellow, and orange indicates the species with a higher percentage of female-biased genes at the indicated cutoff, N is the number of genes. XR passes the significance threshold more often than XL, largely due to a larger number of genes being present on this chromosome, as direction and magnitude of female bias are similar between XL and XR.
(XLSX)

Table S6 Same as table S5, but with the alternate definition of zygotic genes. Zygotic definitions were determined in each species as in Figure 3/Table S5, but genes used were only those classified as zygotic in both species at each stage.
(XLSX)

Table S7 Comparison of sex-bias on autosomes between *D. pseudoobscura* and *D. miranda*. Mean and median female to male ratios are shown for each stage and each species for all major autosomes in both species, Chr. 2 and Chr. 4 (Chr. 3 is the neo-XY in *D. miranda* and thus not an autosome for both species). Wilcoxon test p-values for the comparisons of sex-bias between the species are shown. Yellow indicates significant p-values. *D. miranda* has female-biased autosomes only for the first two stages, where in later stages, both species are close to equal between female and male, with *D. miranda* having a slight male-bias.
(XLSX)

Table S8 Same as table S7, but with the alternate definition of zygotic genes. Zygotic definitions were determined in each species as in Figure 3, Table S5, and Table S7, but genes used were only those classified as zygotic in both species at each stage.
(XLSX)

Table S9 Comparing allele-specific expression from the maternal X chromosome in female and male *D. pseudoobscura* and *D. miranda*. This shows the mean transcript level from the maternal allele for males and females of both species at each stage. 95% bootstrap confidence intervals were calculated for the mean by resampling genes with replacement, taking the mean, and finding the 2.5% and 97.5% quantiles over bootstrap simulations. Highlighted in yellow are those values where the male maternal allele is significantly higher (falls above the 97.5% confidence interval) than the maternal allele in the female. Whether looking over the X chromosomes combined and the autosomes combined (A) or each chromosome separately (B), *D. miranda* is delayed one stage relative to *D. pseudoobscura* as to when males have a significantly higher transcript level from the maternal alleles than do females, which indicates a delay in activation of higher male transcription relative to the female, i.e. the onset of MSL-mediated dosage compensation.
(XLSX)

Table S10 Same as table S9, but with the alternate definition of zygotic genes. Zygotic definitions were determined in each species as in Figure 5 and Table S9, but genes used were only those classified as zygotic in both species at each stage.
(XLSX)

Table S11 Proportion H4K16ac enrichment of compensated and not compensated genes in *D. miranda*. Compensated genes are defined here as those less than 1.5x female biased, uncompensated genes are those with greater than 1.5x female bias (sex-bias is calculated for genes with >5RPKM in both female and male). Mean % of H4K16ac data is from Alekseyenko et al, 2013 [33]. 95% bootstrap confidence intervals were calculated for the mean % H4K16ac over all genes on the chromosome by resampling genes with replacement, taking the mean, and finding the 2.5% and 97.5% quantiles over bootstrap simulations. Values falling below the 2.5% confidence interval for the other species are highlighted in blue, values falling above the 97.5% confidence interval for the other species are highlighted in orange.
(XLSX)

Table S12 Wilcoxon test p-values of sex-bias per chromosome over developmental time, for Figure 6A. Significant differences are in yellow.
(XLSX)
