# Supplementary figures and images for: Sex-Specific Embryonic Gene Expression in Species with Newly Evolved Sex Chromosomes
Source: PLoS Genet. 2014 Feb 13;10(2):e1004159. doi: 10.1371/journal.pgen.1004159 (PMC3923672; doi:10.1371/journal.pgen.1004159)

**A**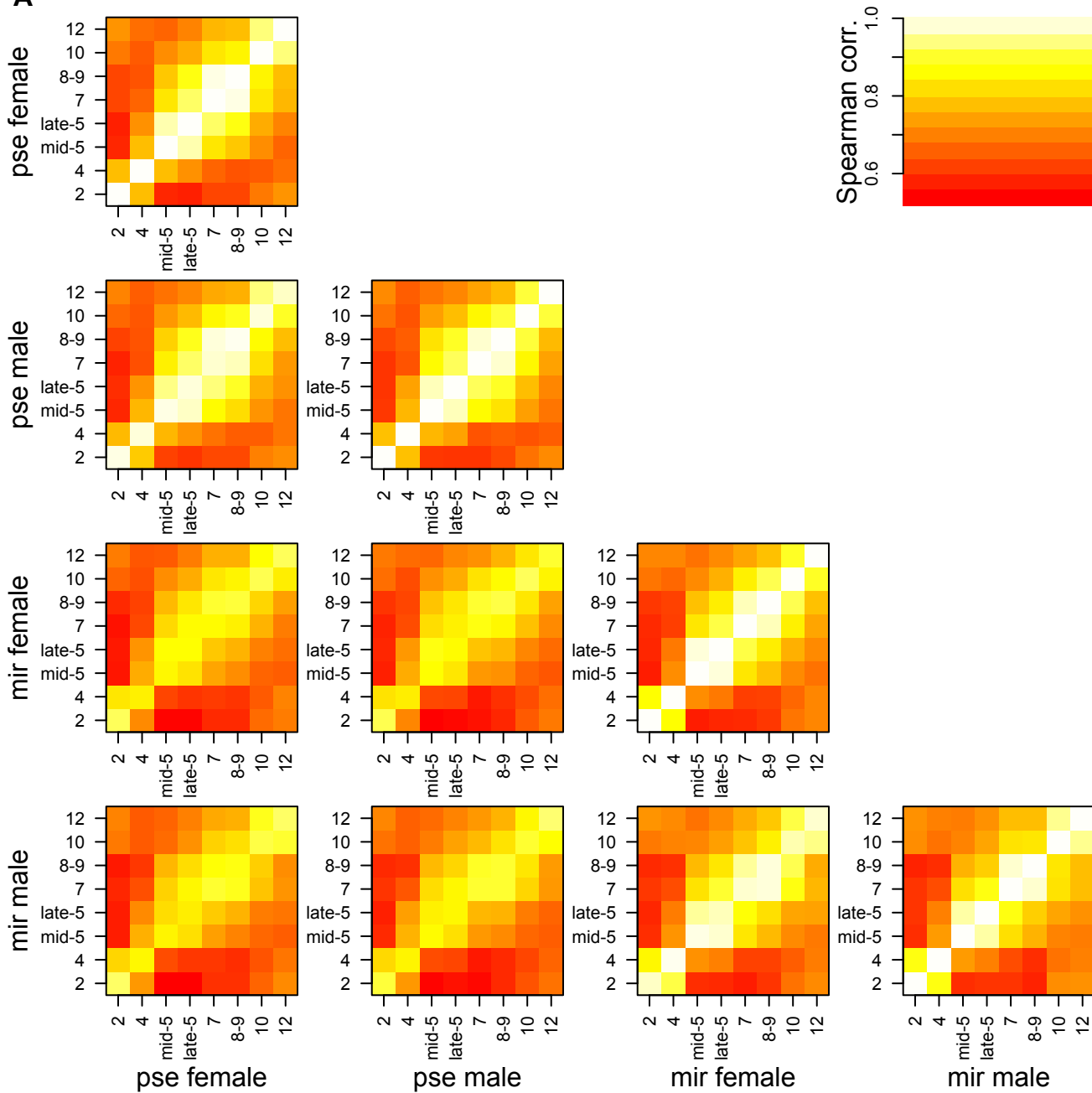

**B**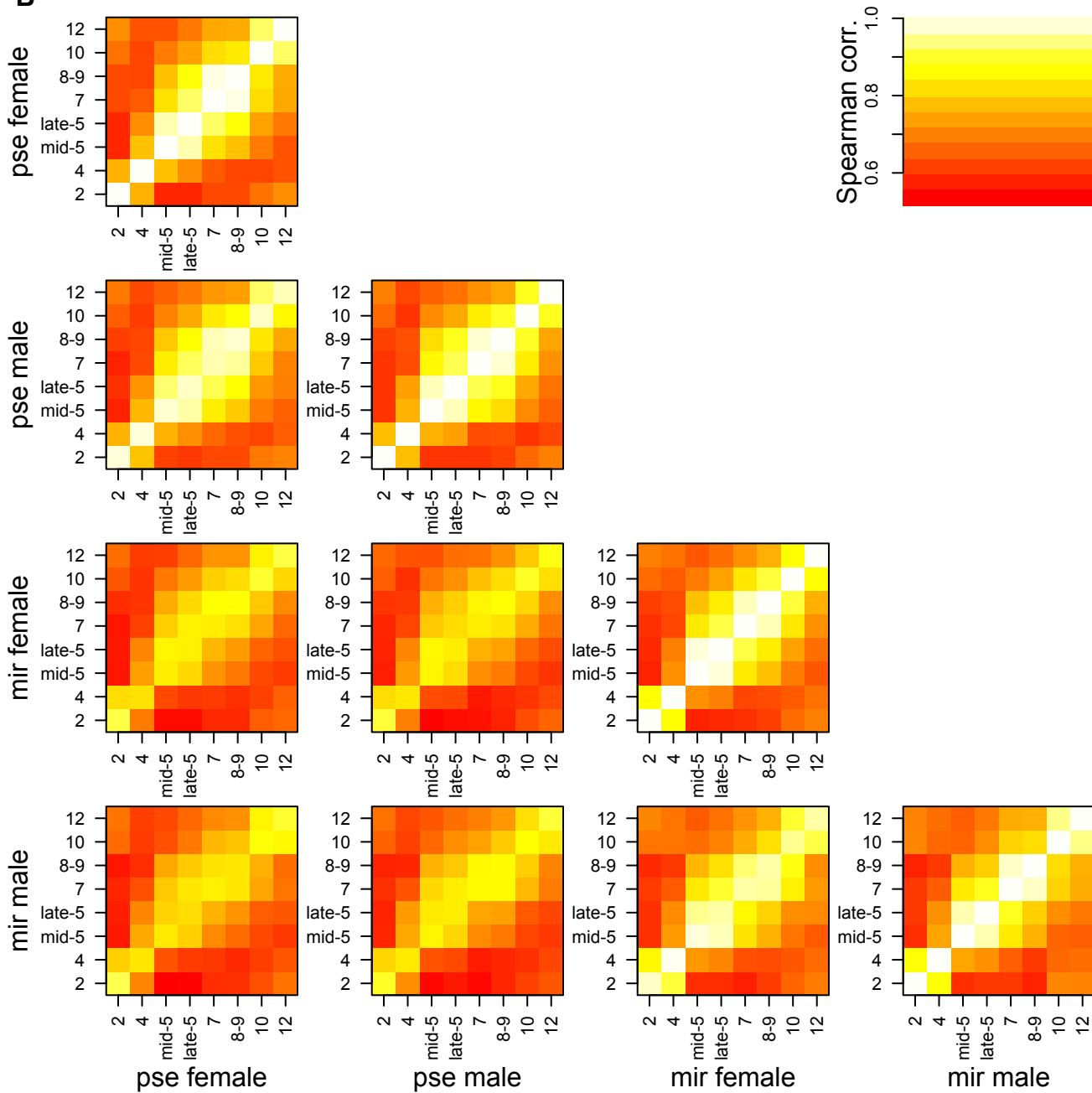

Supplement: Figure S1 — Heatmap of pairwise correlation coefficients between sexes, for mRNA abundance for all genes (in RPKM) at all stages, averaged over all female or male individuals per stage within a species. Both within and between species comparisons are shown. A) shows these plots based on autosomal genes, B) shows these comparisons based on all genes. Statistical analysis shown in Table S4. (PDF) [file pgen.1004159.s001.pdf]

*D. pseudo*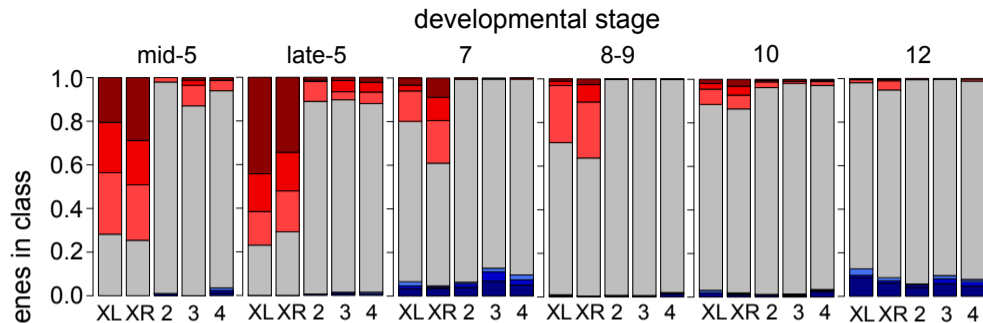*D. miranda*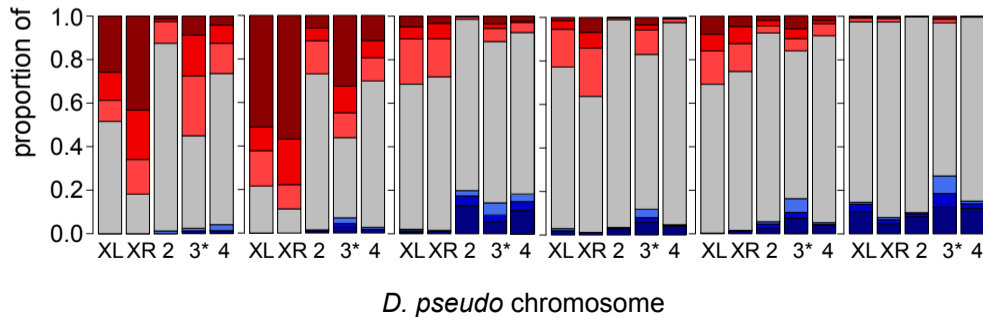

Supplement: Figure S2 — Onset of dosage compensation in D. pseudoobscura and D. miranda, on a genome-wide scale (Figure 3 with an alternate zygotic gene definition). Zygotic definitions were determined in each species as in Figure 3, but genes used were only those classified as zygotic in both species at each stage. Barcharts show proportions of zygotic genes in each sex-bias class, determined by the allele-specific zygotic definition, of female biased (shades of red), male biased (blue), and unbiased genes (grey), for each chromosome. D. pseudoobscura chromosome names were used, so the * is a reminder that this chromosome is the neo-XY in D. miranda, for this chromosome, reads from both the neo-X and neo-Y are included. As roughly half of the genes on the neo-Y are still producing transcript, this chromosome is currently a mix of genes that are hemizygous in males and those that have two functional copies, and perhaps unsurprisingly then, looks better compensated than the ancestral X chromosomes. See Tables S6 and S8 for statistical analyses of the data in this figure. (PDF) [file pgen.1004159.s002.pdf]

# *D. pseudoobscura*

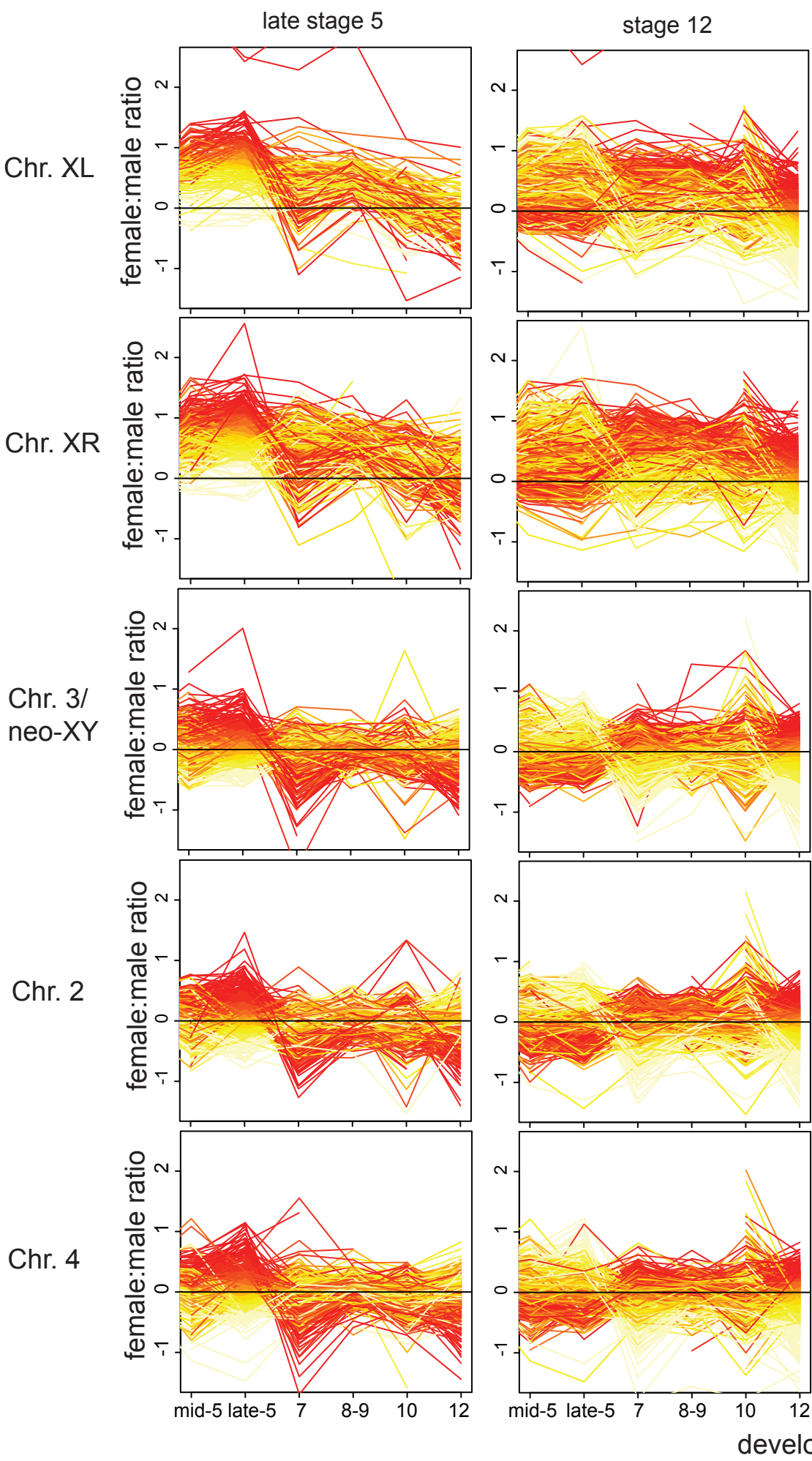

# *D. miranda*

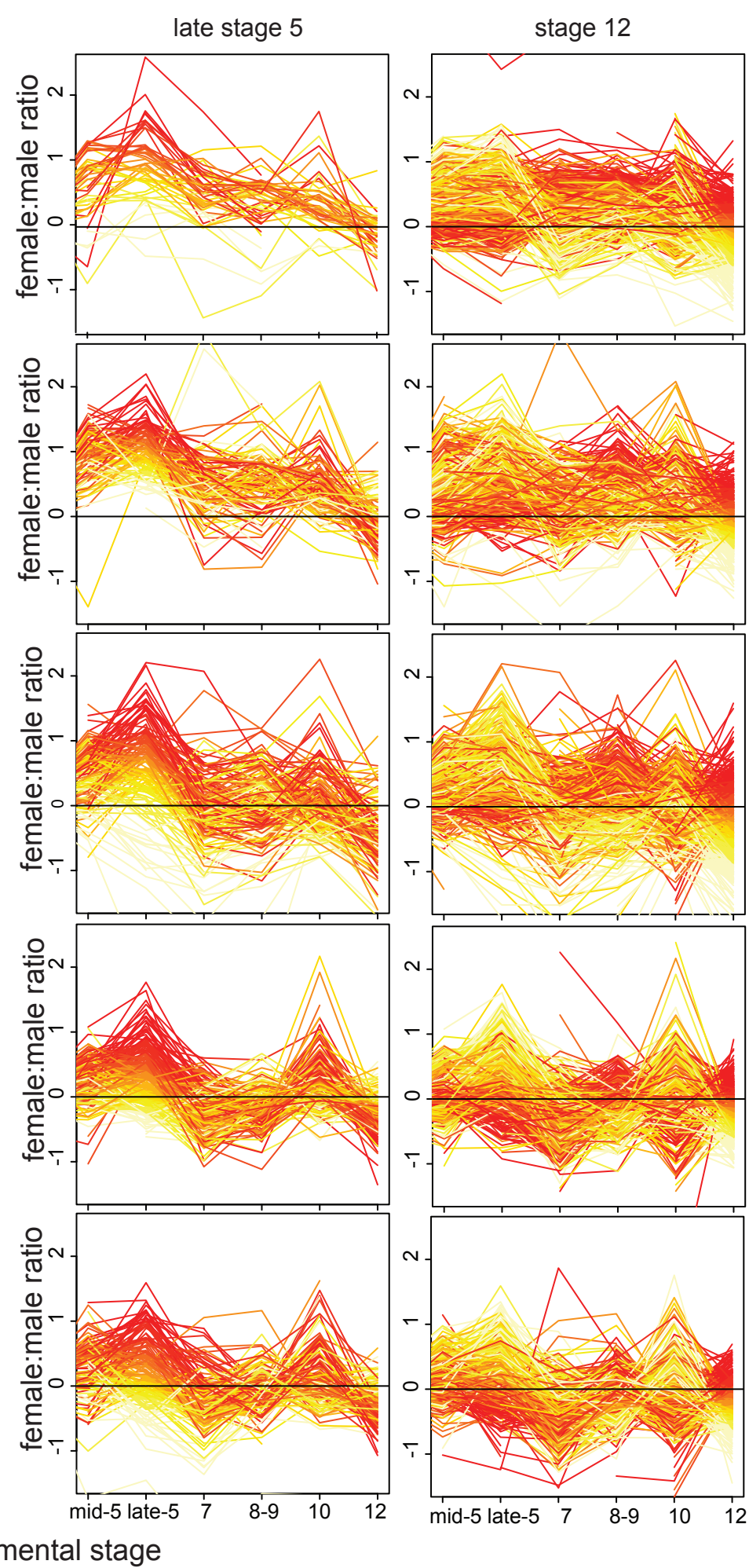

Supplement: Figure S3 — Female to male ratio over stages for all zygotic genes at a particular stage. For a species, the female to male ratio of the zygotic genes at either mid-stage 5 or stage 12 are plotted for the rest of the stages. The color is based on the quantile of the female to male ratio at the stage conditioned on, at the top of the column, with the most female-biased genes in red, least female-biased genes in pale yellow. Female biased genes at early stages (red) are more likely to be less female biased at later stages (yellow), and vice versa, an effect observed most strongly on the autosomes. (PDF) [file pgen.1004159.s003.pdf]

**A**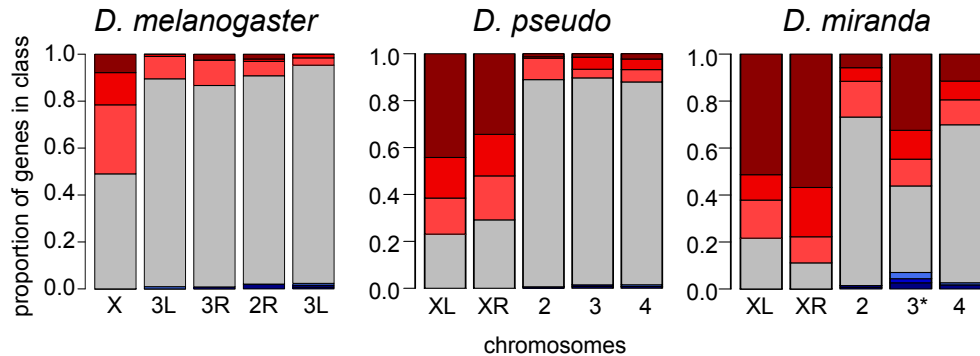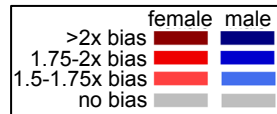**B**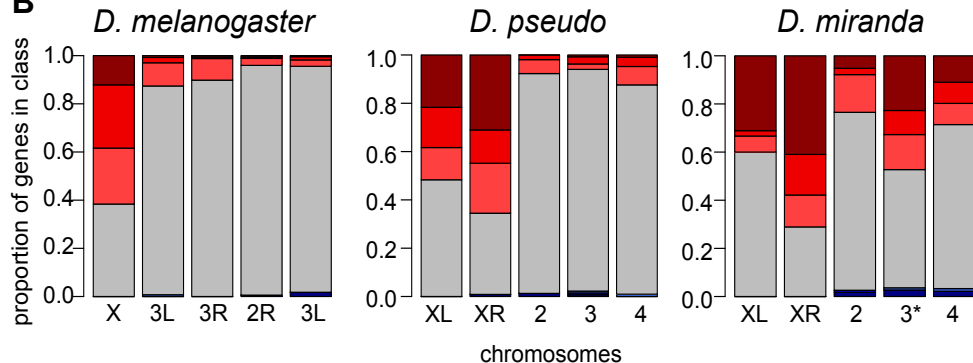

Supplement: Figure S4 — Sex-bias of transcripts in embryo at one timepoint across three species (as in Figure 4) using two alternative zygotic definitions (A & B). Comparison of sex bias in transcripts for zygotically transcribed genes (using the allele-specific zygotic definition) in D. pseudoobscura, D. miranda, and D. melanogaster, at one stage (late stage 5, or the end of blastoderm) early on in development, before the activation of MSL-mediated dosage compensation. Chromosomes are in the same order for all species, D. melanogaster has only the ancestral X, D. pseudoobscura and D. miranda both have XL and XR, and D. miranda additionally has the neo-XY chromosome (indicated with 3*). The neo-XY levels include transcript levels from both the neo-X and the neo-Y in males, and as many of these genes on the neo-Y are still producing transcript in males, this chromosome appears to have less female bias than the ancestral X chromosomes. A) Zygotic definitions were determined in each species as in Figure 4, but the genes used were only those classified as zygotic in both D. pseudoobscura and D. miranda. B) Sex-bias of genes defined as zygotic in D. melanogaster (as in Figure 4), plotted in all species. Using this definition (B), D. melanogaster appears to have similar patterns of sex-bias as in A and Figure 4, whereas D. pseudoobscura and D. miranda have fewer female-biased genes, but still more highly female-biased genes than D. melanogaster. We note in both of these comparisons, genes determined to be zygotically transcribed in one species at this timepoint are not necessarily zygotically transcribed in the other species. Also the numbers of genes are greatly reduced using these gene lists, so results are more likely to be noisy. We were unable to use a shared zygotic gene list across all species using allele-specific zygotic definitions, as this reduced the gene number to just a handful of genes on each chromosome. (PDF) [file pgen.1004159.s004.pdf]

*D. pseudo*  
*D. miranda*

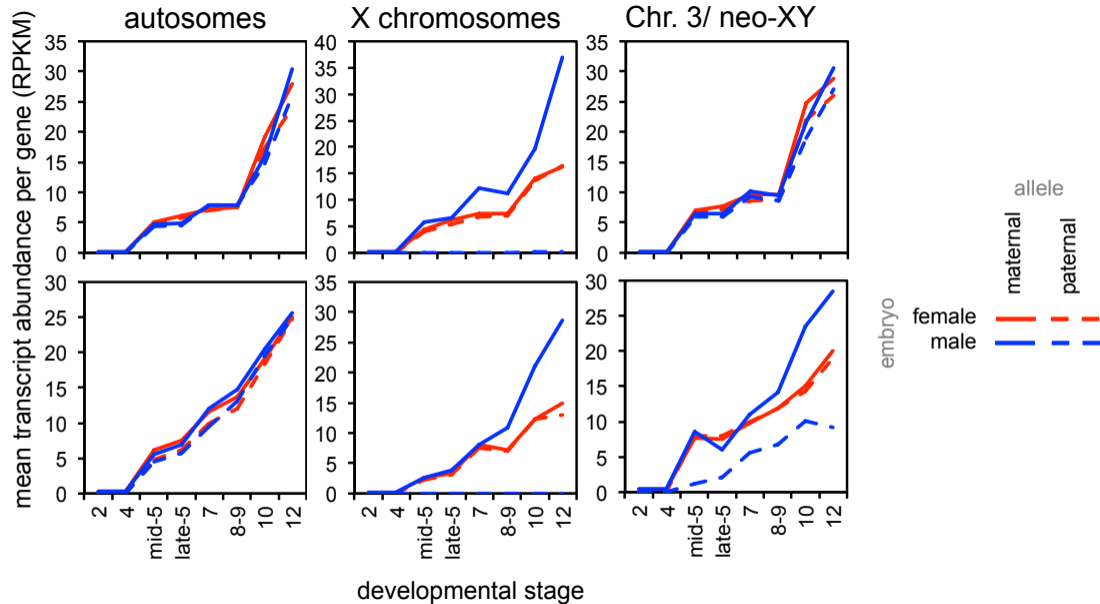

Supplement: Figure S5 — Onset of dosage compensation for zygotically transcribed genes, as revealed by allele-specific expression (as in Figure 5) with alternate zygotic gene definition. Zygotic genes were determined in the same manner for each species as in Figure 5, but only genes that were categorized as zygotic in both species were used in this Figure. Mean proportion of reads per gene attributable to the maternal and paternal alleles, in both female and male embryos, for zygotic genes (defined by transcript level, see Methods). While there are no significant differences in transcript level from the autosomes, the single male X begins to be transcribed at a higher level than either female X, resulting in compensation of chromosome dosage. Unlike D. melanogaster [15], these two species show several stages of zygotic expression with the male X having the same transcript abundance as the female Xs, indicating a period with, on average, no dosage compensation (the D. melanogaster study was of a tighter distribution of stages, and ended at late stage 5). (PDF) [file pgen.1004159.s005.pdf]
